# Acute limb ischemia by a pulmonary vein stump thrombus after left lower lobectomy: a case report

**DOI:** 10.1186/s40981-020-00407-7

**Published:** 2021-01-06

**Authors:** Yuri Fujimoto, Ryosuke Hamachi, Yoshimasa Motoyama, Etsuko Kanna, Masako Murakami, Takako Matsukado, Genkichi Saito, Ken Yamaura

**Affiliations:** 1 Department of Anesthesiology and Critical Care Medicine, Graduate School of Medical Sciences, Kyushu University, 3-1-1 Maidashi, Higashi-ku, Fukuoka, 812-8582 Japan; 2 Department of Anesthesia, Kyushu Central Hospital, 3-23-1, Shiobaru, Minami-ku, Fukuoka, 815-8588 Japan; 3 Department of Surgery, Kyushu Central Hospital, 3-23-1, Shiobaru, Minami-ku, Fukuoka, 815-8588 Japan

**Keywords:** Left lower lobectomy, Pulmonary vein stump thrombus, Acute limb ischemia

## Abstract

**Background:**

Cases of systemic thromboembolism due to thrombus formation in the pulmonary vein stump after lobectomy have been reported recently. Cerebral infarction after left upper lobectomy is a common symptom in these cases. We encountered a rare case of acute limb ischemia caused by a thrombus formed in the left inferior pulmonary vein stump after left lower lobectomy.

**Case presentation:**

A 62-year-old man underwent video-assisted left lower lobectomy under general anesthesia with epidural anesthesia. On postoperative day 2, he suddenly developed pain in the left calf. Contrast-enhanced computed tomography showed left popliteal artery occlusion and thrombus formation in the left inferior pulmonary vein stump. Anticoagulant therapy was started immediately, and emergent endovascular thrombectomy was performed. The patient recovered without complications.

**Conclusions:**

Left lower lobectomy can cause thrombus formation in the pulmonary vein stump, leading to systemic thromboembolism. Early detection and treatment are the keys to minimize complications.

## Background

Systemic thromboembolism due to thrombus formation in the pulmonary vein (PV) stump after lobectomy have been reported recently. Most of these cases occur after left upper lobectomy (LUL), with cerebral infarction as a common symptom [1, 2].

Various vital organs, such as the kidney, intestine, and spleen, are reported to be the target sites of embolism with PV stump thrombosis [3–5]. One case of acute limb ischemia possibly caused by PV stump thrombosis was reported in 1989, and three cases were reported in 2018, all of which were after LUL or left upper division segmentectomy [6, 7].

Cases of embolism caused by a PV stump thrombus are mostly reported from a surgical perspective, and case reports from an anesthetic perspective are relatively rare.

Herein, we report a rare case of acute popliteal artery embolism, probably caused by a thrombus formed in the PV stump after left lower lobectomy (LLL), and give consideration from an anesthetic viewpoint.

## Case presentation

A routine screening chest radiograph of a 62-year-old man (height 177 cm, weight 79 kg) revealed an abnormal shadow in the left basal lung, which was diagnosed as lung carcinoma. He had been treated for diabetes mellitus and coronary spastic angina, which were well-controlled, and was scheduled for video-assisted LLL. He was an active healthcare professional with no history of arrhythmia or thromboembolism. He reported a history of smoking 20 cigarettes a day for 30 years but had quit smoking at the age of 47. A preoperative electrocardiograph showed sinus rhythm, first-degree atrioventricular block, and left anterior hemiblock, but no arrhythmia was detected. Preoperatively, he had a normal platelet count (23.2 × 10^4^/μL) and no coagulation disturbance (prothrombin time international normalized ratio 0.91, activated partial thromboplastin time 27.1 s).

The operation was performed uneventfully under general anesthesia with epidural anesthesia. The operative time was 289 min. The pathological stage was found to be stage I.

**Fig. 1:**
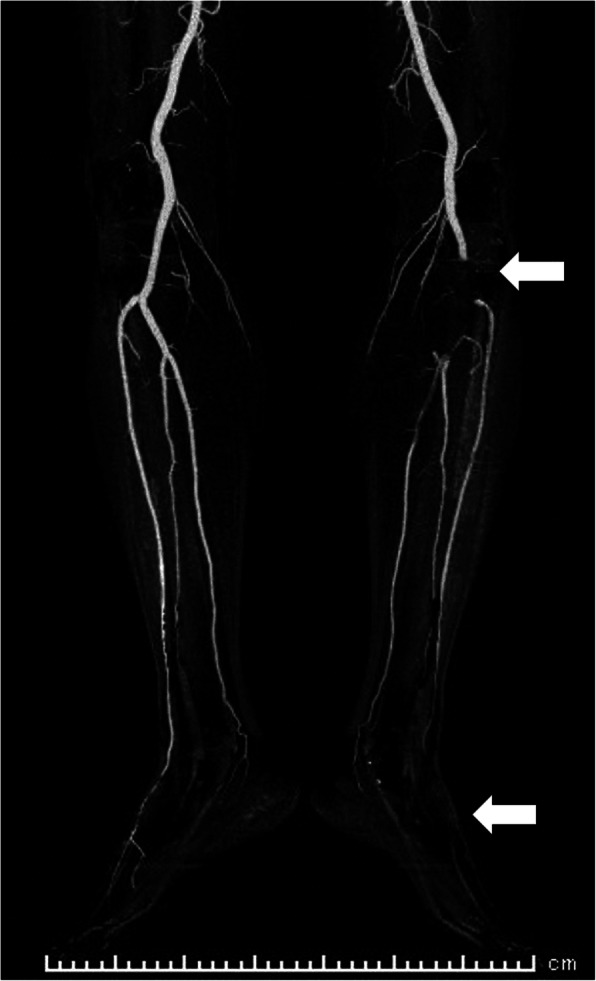
Computed tomography angiography image of the lower extremities taken at acute limb ischemia onset. The upper arrow indicates that the left popliteal artery is occluded, and the lower arrow indicates that the left dorsalis pedis artery cannot be seen clearly

**Fig. 2:**
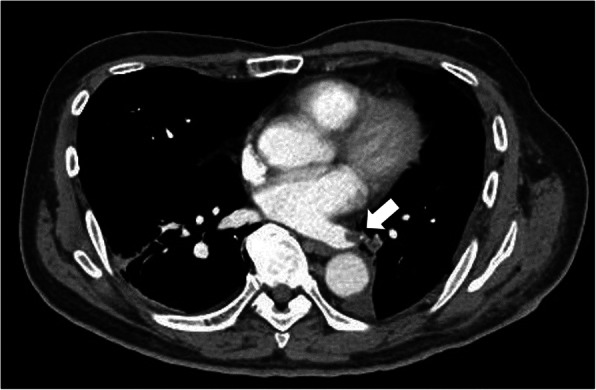
Contrast-enhanced computed tomography image of the chest taken at the onset of acute limb ischemia. The arrow indicates the left inferior pulmonary vein stump thrombus

The patient was ambulatory from postoperative day 1 and had no trouble on that day. On the morning of postoperative day 2, his epidural catheter was removed. He experienced sudden pain in his left calf while going back to bed after brushing his teeth. He complained of this symptom one and a half hours after the epidural catheter removal. His left dorsalis pedis artery was not palpable, but the bilateral femoral and popliteal arteries were palpable, which suggested thrombotic occlusion. A contrast-enhanced computed tomography (CECT) scan, taken an hour after his complaint, showed an occluded left popliteal artery, a poorly described left dorsalis pedis artery (Fig. 1), and thrombus formation in the left lower PV stump (Fig. 2). His blood test revealed an elevated d-dimer level of 3.06 μg/mL. Heparin and alprostadil were started 3 h after the epidural catheter removal, and the patient was transferred to another hospital for endovascular thrombectomy. He had no subsequent complications and was discharged with edoxaban medication. The PV stump thrombus was not found in the CECT scan performed 3 weeks after surgery. Therefore, anticoagulant therapy was stopped.

## Discussion

PV stump thrombus formation after lung resection has been recently studied and recognized as a cause of cerebral infarction and embolism to vital organs. In 2013, Ohtaka et al. found PV stump thrombus on CECT in 3.6% of 193 patients within 2 years after lung lobectomy. Importantly, all patients with a thrombus had undergone left upper lobectomy, and these comprised 13.5% of all patients that underwent left upper lobectomy [1]. A PV stump thrombus other than that of the left superior PV is very rare, and there are only a few reports [8, 9]. The higher frequency of thrombus formation in the left superior PV stump could be because LUL leaves a longer PV stump than other types of lobectomy, which may lead to stagnant blood flow in the PV stump [1].

Dividing the PV to form a PV stump as short as possible has been suggested to prevent PV stump thrombus formation. However, it remains unclear if that will reduce the risk of thrombus formation or thromboembolism; PV stump formation has been reported even when the left superior PV was divided in the pleural space to make the stump as short as possible [10]. If the PV was divided in the pericardium, the PV stump could be even shorter. However, it is not practical to perform this complicated and invasive maneuver in all patients [1].

Prophylactic anticoagulation has also been considered but has not been established because of the risk of bleeding, which can be fatal after lobectomies. Moreover, PV stump thrombosis can occur with anticoagulant therapy [11]. Reported durations from lung resection to PV stump thrombus confirmation or arterial embolism occurrence vary from 1 day to 7 years [9]. This suggests that further investigation is needed to determine the appropriate period and duration for prophylactic anticoagulation. AF causes thrombus formation by blood stagnation in the left atrium; the risk of thromboembolic complication is very low when cardioversion is performed within 48 h of AF onset [12]. Conversely, a recent study has shown that cerebral infarction after pulmonary lobectomy tends to occur very early during the postoperative phase. Hattori et al. reported that 60% of their 10 cases of cerebral infarction after pulmonary lobectomy or more extensive intervention occurred within 2 postoperative days [2]. Similarly, in our case, thromboembolism occurred within 48 h of the operation. This indicates that factors other than blood stagnation may cause PV stump thrombus-related embolism. According to the pathological findings, not only blood stagnation, but also inflammation at the PV stump possibly contributes to PV stump thrombus formation [9].

Left upper lobectomy, operative time, elderly age, and advanced stages of lung cancer (pathological stage II or more) have been indicated as risk factors for PV stump thrombus formation [1, 2]. Elderly age may be related to the fragility of the endothelium, while the advanced stage of lung cancer may be related to a hyper-thrombotic state.

From an anesthetic viewpoint, Kitajima et al. reported the implementation of routine intravenous systemic heparinization following LUL after encountering a patient who developed cerebral infarction post-LUL. Along with this postoperative anticoagulant therapy, they changed the postoperative analgesia from epidural analgesia to intercostal nerve blocks with intravenous patient-controlled analgesia [13]. In this way, other methods of postoperative analgesia, such as continuous nerve blocks, may be utilized instead of epidural anesthesia if the risk factors for PV stump thrombus formation and prophylactic anticoagulation are established in the future. Therefore, it is important that these rare cases be reported.

This case provides crucial evidence linking LLL, thrombus formation in the pulmonary vein stump, and systemic embolism. It is important for medical caregivers to recognize that embolism caused by a PV stump thrombus may occur in lobectomies other than LUL. The patient may present with varied symptoms, as the target site may be systemic. Since the prophylactic prevention of PV stump thrombus formation has not been established thus far, early detection and treatment are the keys to minimize complications. Anesthesiologists should be aware of this complication to detect it as early as possible if it happens and to seek the safe and effective methods of postoperative analgesia.

## Data Availability

Data relevant to this case report are not available for public access because of patient privacy concerns, but are available from the corresponding author on reasonable request.

## Abbreviations

PV: Pulmonary vein
LUL: Left upper lobectomy
LLL: Left lower lobectomy
CECT: Contrast-enhanced computed tomography
AF: Atrial fibrillation
